# Polysomnography Indicators Associated With Moderate Depression: A Cross-Sectional Study

**DOI:** 10.31083/AP52813

**Published:** 2026-10-10

**Authors:** Qianqi Zhao, Junhe Zhou, Junjun Chen, Wenbin Fu

**Affiliations:** ^1^The Second Clinical Medical College, Guangzhou University of Chinese Medicine, 510006 Guangzhou, Guangdong, China; ^2^Department of Acupuncture and Moxibustion, The Second Affiliated Hospital of Guangzhou University of Chinese Medicine (Guangdong Provincial Hospital of TCM), 510120 Guangzhou, Guangdong, China; ^3^The First Child and Adolescent Psychiatry Department, Chongqing Eleventh People’s Hospital, 400030 Chongqing, China

**Keywords:** moderate depressive disorder, polysomnography, sleep quality, multivariate binary logistic analysis, ROC curve

## Abstract

**Background:**

This study aimed to use polysomnography (PSG) and clinical rating scales to evaluate sleep quality, sleep characteristics, and their associations in patients with moderate depression.

**Methods:**

A total of 52 patients with moderate depression and 21 healthy controls were enrolled. All participants underwent PSG and were assessed using the Pittsburgh Sleep Quality Index (PSQI). Differences in clinical characteristics between the two groups were analyzed. Multivariate binary logistic regression analysis was performed to identify factors associated with moderate depression. Receiver operating characteristic (ROC) curve analysis was used to evaluate the diagnostic performance of PSG indicators for moderate depression.

**Results:**

Compared with healthy controls, patients with moderate depression exhibited significantly higher total PSQI scores and poorer objective sleep quality based on PSG measurements. Specifically, they had shorter total sleep time (TST), longer wake after sleep onset (WASO), lower sleep efficiency (SE), a higher percentage of wakefulness (W%), fewer rapid eye movement (REM) sleep cycles, and a higher apnea-hypopnea index (AHI) (all *p* < 0.05). Multivariate binary logistic regression analysis showed that each 1-point increase in the PSQI score was associated with a 6.66-fold increase in the odds of moderate depression (95% CI = 1.143–38.835, *p* = 0.035). The PSQI score showed excellent discriminative performance (AUC = 0.990, 95% CI: 0.974–1.000), whereas the univariate ROC results for age, ArI, REM cycle, and AHI should be interpreted cautiously as exploratory findings rather than evidence of independent predictive value.

**Conclusions:**

Patients with moderate depression exhibit poorer subjective and objective sleep quality than healthy individuals and demonstrate distinct polysomnographic characteristics.

## Main Points

1. Patients with moderate depression exhibited poorer sleep quality and distinct polysomnography (PSG) features compared with healthy controls, highlighting significant differences in sleep-related aspects between the two groups.

2. Multivariate binary logit model regression analysis revealed that the Pittsburgh Sleep Quality Index (PSQI) was a relevant factor for moderate depression, providing insights into the factors influencing moderate depression.

3. Receiver operating characteristic (ROC) curve analysis showed that age, PSQI value, arousal index (ArI), rapid eye movement (REM) cycle, and apnea-hypopnea index (AHI) were independently associated with moderate depression, with specific area under the ROC curve (AUC) values and optimal cut-off values. AHI was associated with moderate depression and can therefore be used to assist in the early identification and risk assessment of this condition.

4. Although not statistically significant, the moderate depression group showed a decrease in REM sleep, while the depression group unexpectedly had slightly higher N3 sleep.

## 1. Introduction

Depressive disorder imposes a substantial global health burden, with a rising prevalence among younger populations [1]. According to the World Health Organization, depression is projected to become the leading cause of disease burden worldwide by 2030 [2,3]. In China, the pooled prevalence of depressive symptoms among Chinese children and adolescents is 26.17% [4]. In addition to personal suffering, families often face financial pressures, including long-term treatment costs and reduced family income due to the absence of the caregiver [2]. The intergenerational transmission of depressive symptoms increases the risk of mental illness in offspring [5]. Studies have shown that the majority of affected individuals experience social dysfunction, yet the utilization of health services and receipt of adequate treatment remain low [6]. As a chronic condition with a high risk of recurrence across the lifespan, depression is associated with considerable disability, economic costs, and an elevated risk of suicide. Early identification of depressive onsets is therefore of critical clinical importance.

Large-scale genome-wide association studies have confirmed that depression is highly polygenic, with common variants jointly explaining a substantial proportion of risk variation [7]. Among adolescent girls, depressive symptoms, rumination, parental mood disorders, and parental criticism were independently associated with first-episode depression [8]. Moreover, cognitive behavioral prevention has been shown to predict and alleviate depressive episodes in high-risk adolescents [9]. However, these predictors were largely assessed through interviews or subjective rating scales, highlighting the need for objective monitoring approaches in clinical settings. Given that about half of patients diagnosed with major depressive disorder have an inadequate response to existing treatments [10], there is growing interest in identifying biomarkers that can predict the disease course and distinguish between treatment responders and nonresponders [11,12].

Polysomnography (PSG) is considered the gold standard for objective evaluation in sleep medicine [13]. Compared with subjective sleep indicators, PSG directly captures physiological signals and can detect clinically unrecognized abnormal events [14]. It provides cross-center and cross-time point comparative indices that offer a unique quantitative basis for the objective diagnosis and accurate classification of sleep disorders and facilitate the assessment of sleep characteristics associated with depression [15,16]. Wearable sleep-tracking devices are being increasingly adopted in the field of sleep and circadian rhythm [17]. Patients with depression exhibit characteristic alterations in sleep architecture on PSG, and accumulating evidence suggests that various sleep electroencephalography (EEG) parameters may serve as biomarkers for depression diagnosis, prognosis, and treatment-response prediction [15,18]. Sleep disturbances have recently been identified as the most frequent prodromal symptom preceding depressive episodes [19]. Given that sleep disorders affect almost a quarter of the global population and are highly comorbid with mental illness [20], the Diagnostic and Statistical Manual of Mental Disorders, Fifth Edition (DSM-5) already includes sleep disturbances—such as insomnia, early-morning awakening, or hypersomnia—among the diagnostic criteria for depression [21]. This bidirectional association has reframed sleep problems not merely as an epiphenomenon of depression, but rather as a potential prodromal indicator warranting independent clinical attention [22].

In light of this relationship, the examination of depression through the lens of sleep architecture holds considerable promise. Importantly, depression severity may modulate sleep quality [23], with moderate depression imposing notable functional impairments. Nevertheless, previous studies have predominantly focused on severe depression or insomnia–depression comorbidity, and evidence that specifically addresses moderate depression remains limited [24]. This gap is critical because subjective sleep measures lack the resolution to detect subtle architectural alterations that may distinguish moderate depression from healthy controls. Accordingly, the present study focused on patients with moderate depression and utilized PSG to systematically characterize their sleep profile indicators with the aim of informing early identification and intervention.

## 2. Materials and Methods

### 2.1 Participants

The sample consisted of 52 patients with moderate depression and 21 healthy controls, recruited at the Second Affiliated Hospital of Guangzhou University of Chinese Medicine from January 2023 to January 2024.

All participants with moderate depression met the following criteria: no use of antipsychotic, antidepressant, sedative, or hypnotic drugs within 2 weeks before enrolment; and no history of alcohol abuse or major illness. In addition, these patients met DSM-5 (American Psychiatric Association, 2013) criteria and had a 17-item Hamilton Depression Rating Scale (HAMD-17) score between 18–24 inclusive.

The control group was matched with the moderate depression group in terms of age, gender, height, and weight. The subjects were confirmed to be in good health by physical examination, and had no important organic, mental, or functional diseases. The exclusion criteria were: (1) recent fever and infection; (2) use of psychotropic or sedative-hypnotic drugs in the past 3 months; (3) recent heavy drinking.

The age of participants in both groups ranged from 18 to 65 years. The study was carried out in accordance with the guidelines of the Declaration of Helsinki, and the protocol was approved by the Ethics Committee of the Second Affiliated Hospital of Guangzhou University of Chinese Medicine (approval number: BF2020-186-02). Written informed consent was obtained from all participants at the initial visit prior to enrolment.

### 2.2 Polysomnography (PSG)

An overnight PSG was performed for each participant using an Embla N700 system (MDrive REF: 2000105, Kanata, Ottawa, Ontario, Canada). Recording sessions typically commenced at 22:00 and concluded at 07:00 the following morning, with a minimum recording duration of 7 hours. Following biocalibration, peripheral oxygen saturation (SpO_2_) was recorded. Raw data were automatically stored by the system. Sleep data were manually scored in 30-second epochs by a trained scorer who was blinded to group assignment, in accordance with the American Academy of Sleep Medicine (AASM) Manual for the Scoring of Sleep and Associated Events (version 2.6). Sleep stages were classified as N1, N2, N3, and rapid eye movement (REM) sleep. Based on the scoring, the following parameters were computed: total sleep time (TST), wake after sleep onset (WASO), sleep efficiency (SE), REM sleep latency, the percentage of each sleep stage (wake [W%], N1%, N2%, N3%, REM%), arousal index (ArI), sleep latency (SL), slow-wave sleep (SWS), periodic limb movements during sleep (PLMS), apnea-hypopnea index (AHI), and mean heart rate.

### 2.3 Hamilton Depression Scale (HAMD-17)

Depressive symptoms were measured using the Hamilton Depression Rating Scale (HAMD-17) [25] on the same night that PSG was conducted. Two trained assessors independently evaluated each participant, and the final score was determined by consensus. The total score for HAMD-17 in this study ranged from 0 to 52 (higher scores indicate greater symptom severity). Depressive severity was classified as follows: mild depression, 7 < score ≤ 16; moderate depression, 17 < score ≤ 23; severe depression, score ≥24. Scores ≤7 indicated no depression [26].

### 2.4 Pittsburgh Sleep Quality Index (PSQI)

The Pittsburgh Sleep Quality Index (PSQI) [27] was administered to evaluate sleep quality over the preceding month. This 19-item questionnaire yields seven component scores (each 0–3), with the sum of these components giving a total score ranging from 0 to 21. Higher total scores indicate worse sleep quality.

### 2.5 Statistical Analysis

Participants were divided into two groups based on the presence or absence of moderate depression. Demographic and clinical variables were summarized and compared between the two groups. The normality of continuous variables was assessed using the Shapiro–Wilk test. Normally distributed continuous variables were presented as the mean ± standard deviation (SD), and non-normally distributed variables as the median (P25, P75). Between-group comparisons were performed using the independent-samples *t*-test (for normally distributed data) or Mann–Whitney U test (for non-normally distributed data). Categorical variables were expressed as numbers (percentages) and compared using the chi-square test or Fisher’s exact test, as appropriate.

To examine associations between moderate depression and clinical rating scales as well as PSG parameters, Spearman correlation analysis was used for non-normally distributed variables. For causal inference, binary logistic regression analysis was performed to identify independent factors associated with moderate depression.

A predictive model was then constructed based on the independent factors identified. The discriminative ability of the model was evaluated using receiver operating characteristic (ROC) curves, and the area under the ROC curve (AUC) was calculated. An AUC >0.7 was considered to indicate acceptable discrimination. The optimal cut-off value was determined based on the maximum Youden index.

All statistical analyses were performed using SPSS version 27.0 (IBM Corp., Armonk, NY, USA) and R (version 4.2.2; R Foundation for Statistical Computing, Vienna, Austria). A two-tailed *p*-value < 0.05 was considered statistically significant. Given that multiple PSG variables were tested, we applied the Benjamini-Hochberg false discovery rate (FDR) correction to control for multiple comparisons. An FDR-adjusted *q*-value < 0.05 was considered significant.

## 3. Results

### 3.1 Demographic Characteristics

Demographic characteristics of the two groups are presented in Table 1. A total of 73 participants were included: 52 patients with moderate depression (71.2%) and 21 healthy controls (28.8%). Of these, 8 were male (11.0%), and 65 were female (89.0%). No statistically significant differences were observed between the two groups in gender, height, or weight (all *p* > 0.05).

**Table 1.** **Comparison of demographic data between the two groups**.

Variables	Healthy individuals (n = 21)	Moderate depression (n = 52)	*Z/χ^2^*	*p*
Age, M (P25, P75), years	25.9 (25.3, 28.4)	28.7 (25.6, 31.2)	–2.096	0.036
Gender: male, n (%)	2 (9.5%)	6 (11.5%)	0.096	0.557
Gender: female, n (%)	19 (90.5%)	46 (88.5%)		
Height, M (P25, P75), cm	158.5 (155.0, 164.3)	160.0 (156.3, 165.8)	–0.939	0.348
Weight, M (P25, P75), kg	54.5 (47.8, 58.0)	52.5 (45.3, 66.0)	–0.521	0.603

Note: M, median.

### 3.2 Sleep Quality of Patients With Moderate Depression vs Healthy Individuals

Comparisons of PSQI scores and PSG parameters between patients with moderate depression and healthy controls are presented in Table 2. Corrections for multiple comparisons were made using the Benjamini-Hochberg FDR method (*m* = 18). After FDR correction, 8 of 18 variables remained statistically significant (*q* < 0.05).

**Table 2.** **Comparison of total scores for the PSQI and PSG indicators between the two groups**.

Variables	Healthy individuals (n = 21)	Moderate depression (n = 52)	*t/Z*	*p*	*q* (FDR-adjusted* p*)
PSQI, mean (SD)	3.62 ± 1.59	12.35 ± 3.56	–6.544	<0.001	0.006
TST, M (P25, P75), min	515.5 (501.5, 558.0)	490.5 (436.9, 529.9)	–2.773	0.006	0.015
WASO, M (P25, P75), min	43.5 (20.5, 79.5)	89.3 (48.1, 139.5)	–3.132	0.002	0.007
SE, M (P25, P75), %	91.7 (86.4, 96.0)	81.6 (70.7, 89.2)	–3.516	<0.001	0.006
REM, M (P25, P75), %	25.5 (20.6, 27.0)	19.3 (14.4, 23.4)	–2.243	0.025	0.0500
N1, M (P25, P75), %	9.9 (7.7, 11.2)	8.9 (6.6, 14.2)	–0.469	0.639	0.767
N2, M (P25, P75), %	54.1 (46.9, 57.1)	54.0 (47.1, 61.5)	–0.104	0.917	0.917
N3, M (P25, P75), %	10.6 (9.5, 15.6)	13.4 (10.5, 20.5)	–2.084	0.037	0.067
W, M (P25, P75), %	16.3 (9.1, 25.9)	28.2 (17.9, 43.9)	–3.875	<0.001	0.006
ArI, M (P25, P75), times/h	1.8 (1.2, 2.2)	2.8 (1.9, 3.8)	–3.108	0.002	0.007
SL, M (P25, P75), min	24.5 (13.0, 62.0)	47.3 (19.6, 97.6)	–1.840	0.066	0.108
REM latency, M (P25, P75), min	121.0 (75.5, 150.5)	100.0 (67.3, 172.9)	–0.524	0.6	0.767
REM cycle, M (P25, P75)	4.0 (4.0, 5.0)	3.0 (2.0, 4.8)	–2.723	0.006	0.015
SWS cycle, M (P25, P75)	3.0 (2.0, 3.0)	3.0 (2.0, 4.0)	–0.528	0.598	0.767
PLMS, M (P25, P75), times/h	0.0 (0.0, 1.5)	0.0 (0.0, 2.2)	–0.220	0.826	0.917
AHI, M (P25, P75), times/h	2.2 (1.7, 3.9)	3.6 (2.3, 6.2)	–2.383	0.017	0.038
SpO_2_, M (P25, P75), %	95.7 (93.9, 96.9)	95.6 (94.7, 96.7)	–0.134	0.893	0.917
Average heart rate, mean (SD), beats/min	64.90 ± 5.98	62.69 ± 7.30	–1.423	0.155	0.233

Note: PSQI, Pittsburgh Sleep Quality Index; TST, total sleep time; WASO, wake after sleep onset; SE, sleep efficiency; REM, rapid eye movement; W, N1, N2, N3, percentage of each sleep stage; ArI, arousal index; SL, sleep latency; SWS, slow wave sleep; PLMS, periodic limb movements of sleep; AHI, apnea–hypopnea index; SpO_2_, mean peripheral oxygen saturation.

Patients with moderate depression had significantly higher PSQI scores than healthy controls (*q* = 0.006). For PSG parameters, the moderate depression group had significantly worse performance in several time-related metrics, including TST, WASO, and SE (all *q* < 0.05). Significant between-group differences were also observed in W%, ArI, and REM cycle (all *q* < 0.05). Furthermore, the AHI was significantly higher in the moderate depression group compared with controls (*q* = 0.038). No significant between-group differences were found in SpO_2_ or mean heart rate (both *q* > 0.05).

### 3.3 Results of Correlation Analysis

To address the potential overlap between HAMD-17 and PSG measures, correlation analyses were performed among HAMD, PSQI, and PSG parameters in the moderate depression group after excluding the three sleep-related HAMD items (item 4: early insomnia; item 5: middle insomnia; item 6: late insomnia) (Table 3). The sensitivity analysis excluding HAMD sleep items (items 4–6) revealed no significant correlations between HAMD-14 scores and any PSG parameters (all *q* > 0.05). Similarly, no significant correlation was observed between PSQI and HAMD-14 scores (*q* > 0.05). These findings suggest the previously observed HAMD–PSG and PSQI–HAMD associations were largely driven by shared sleep-related content rather than reflecting a direct link between depression severity and objective sleep architecture.

**Table 3.** **Spearman Correlation analysis between moderate depression and subjective and objective sleep indicators**.

	HAMD score		
Variable	*r*	*p*	*q *(FDR-adjusted *p*)
PSQI score	–0.001	0.994	0.994
TST, min	0.179	0.203	0.466
WASO, min	–0.144	0.308	0.554
SE, %	0.195	0.166	0.466
REM, %	0.134	0.345	0.565
N1, %	–0.101	0.478	0.615
N2, %	0.168	0.233	0.466
N3, %	–0.386	0.005	0.090
W, %	–0.288	0.038	0.228
ArI, times/h	0.078	0.581	0.697
SL, min	–0.253	0.071	0.295
REM latency, min	0.050	0.725	0.785
REM cycle	0.243	0.082	0.295
SWS cycle	–0.322	0.020	0.180
PLMS, times/h	–0.173	0.219	0.466
AHI, times/h	0.101	0.474	0.614
SpO_2_, %	0.122	0.390	0.585
Average heart rate, beats/min	0.047	0.741	0.785

### 3.4 Multivariate Binary Logistic Regression Analysis

Multicollinearity among predictors was assessed using variance inflation factors (VIF) and tolerance values. Variables with a VIF ≥4 were considered indicative of problematic multicollinearity. As shown in Table 4, TST (VIF = 6.584), WASO (VIF = 78.439), SE (VIF = 58.035), and W% (VIF = 14.550) exhibited severe multicollinearity. To ensure model stability, TST, WASO, SE, and W% were excluded from the final logistic regression model.

**Table 4.** **Collinearity analysis**.

Variable	Non-standardization coefficient		*t*	*p*	Collinearity statistics	
	*β*	*β *Standard error			Tolerance	VIF
Gender	–0.043	0.11	–0.387	0.700	0.949	1.054
Age, y	<0.001	0.005	–0.004	0.997	0.817	1.224
PSQI score	0.063	0.008	7.678	<0.001	0.655	1.527
TST, min	–0.002	0.001	–1.302	0.198	0.152	6.584
WASO, min	0.001	0.004	0.374	0.710	0.013	78.439
SE, %	0.001	0.017	0.042	0.967	0.017	58.035
W, %	–0.003	0.008	–0.408	0.685	0.069	14.55
ArI, times/h	–0.002	0.019	–0.103	0.918	0.541	1.849
REM cycle	–0.006	0.024	–0.259	0.796	0.543	1.841
AHI, times/h	0.007	0.011	0.649	0.519	0.908	1.102

Note: VIF, variance inflation factor. A VIF <4 indicates a well-constructed model with no multicollinearity issues.

Multivariate logistic regression analysis was performed on indicators with statistically significant between-group differences. The results showed that for each 1‑unit increase in the PSQI score, the odds of depression were 6.663 times higher (OR = 6.663, 95% CI = 1.143–38.835, *p* = 0.035). As shown in Table 5, no significant associations were observed between depression and gender, age, ArI, REM cycle, or AHI (all *p* > 0.05).

**Table 5.** **Multivariate regression analysis of sleep time in patients with moderate depression**.

Variables	*β*	Standard error	Wald	*p*	OR	95% CI	
						Lower CI [2.5%]	Upper CI [97.5%]
Gender	0.142	7.131	0	0.984	1.153	0	1,354,515.148
Age, years	0.402	0.231	3.03	0.082	1.495	0.951	2.353
PSQI score	1.897	0.899	4.447	0.035	6.663	1.143	38.835
ArI, times/h	0.715	1.264	0.32	0.572	2.045	0.172	24.37
REM cycle	–0.520	0.971	0.287	0.592	0.594	0.089	3.989
AHI, times/h	0.862	0.841	1.05	0.305	2.368	0.455	12.308

### 3.5 Results of ROC Analysis

ROC curve analysis was conducted using gender, age, and PSG indices with VIF <4 as test variables to evaluate their diagnostic performance in distinguishing patients with moderate depression from healthy controls. The PSQI score showed excellent discriminative performance (AUC = 0.990, 95% CI: 0.974–1.000). Other variables, including age, ArI, REM cycle count, and AHI, exhibited lower or reversed AUC values (range 0.299–0.734). It is important to note that after adjusting for other factors in multivariate logistic regression, only the PSQI score remained significantly associated with moderate depression (Table 5). Therefore, the univariate ROC results for age, ArI, REM cycle, and AHI should be interpreted cautiously as exploratory findings rather than evidence of independent predictive value (Table 6 and Fig. 1).

**Table 6.** **Models for assessing the risk of moderate depression using PSG indicators**.

Variable	AUC	95% CI		Cut-off	Sensitivity (%)	Specificity (%)	Youden index	*p*
		Lower CI [2.5%]	Upper CI [97.5%]					
Gender	0.490	0.344	0.636	0.000	100.00	0.000	0.000	0.893
Age, y	0.658	0.533	0.782	29.016	50.000	50.000	0.405	0.036
PSQI score	0.990	0.974	1.000	6.500	98.100	1.900	0.933	<0.001
ArI, times/h	0.734	0.612	0.855	2.105	69.200	30.800	0.406	0.002
REM cycle	0.299	0.179	0.419	6.500	3.800	96.200	0.038	0.007
AHI, times/h	0.679	0.549	0.809	2.650	75.000	25.000	0.321	0.017

Note: AUC, area under curve.

**Fig. 1.**
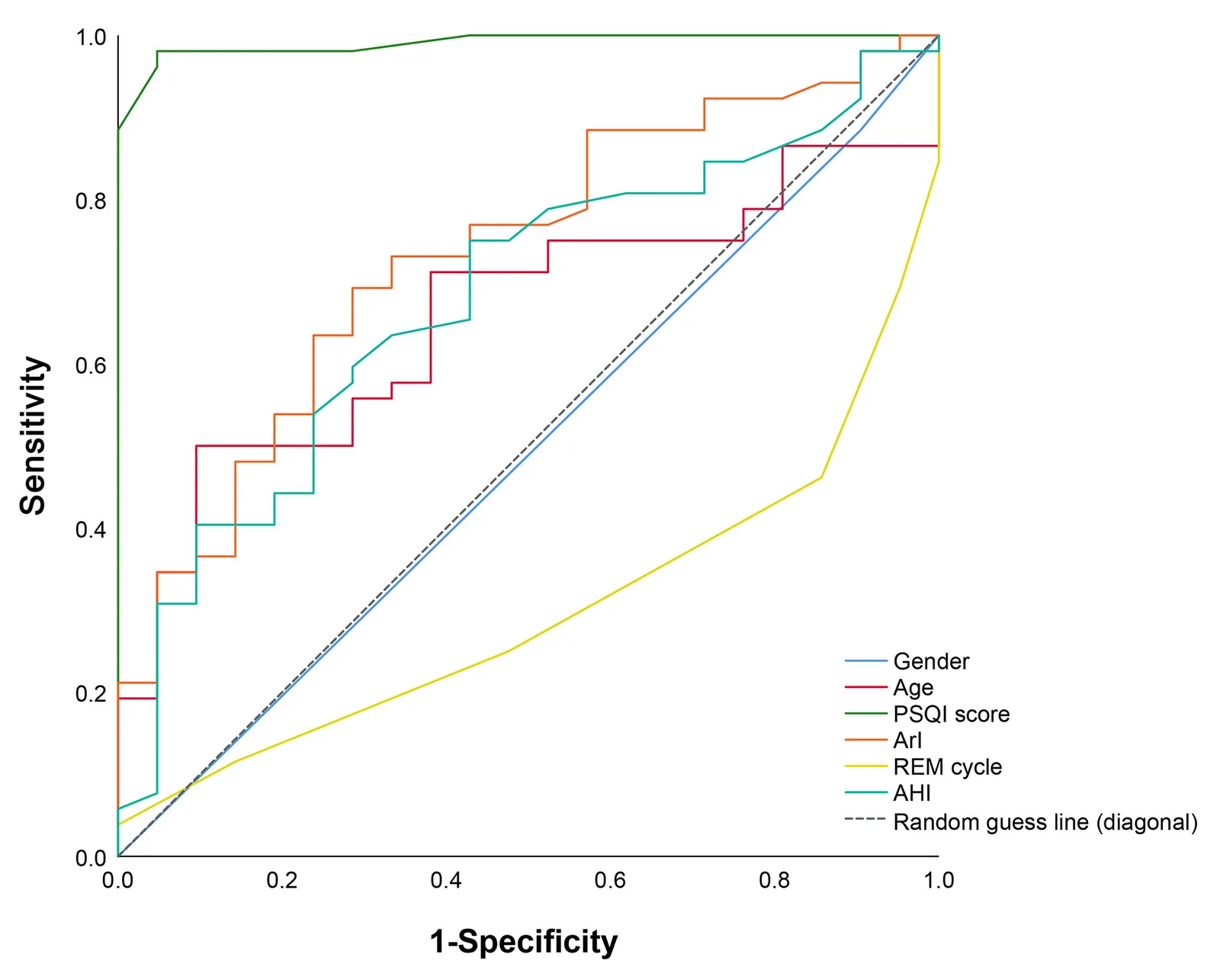
**ROC curves of individual predictors**.

## 4. Discussion

This study investigated differences in sleep architecture between patients with moderate depressive disorder and healthy controls using PSG and the PSQI scale. Our results showed that patients with moderate depressive disorder exhibited significantly poorer sleep quality on both subjective and objective measures. The depression group had significantly higher PSQI scores, shorter total sleep time, lower sleep efficiency, longer wake after sleep onset, more frequent awakenings, higher arousal index, a tendency toward reduced REM sleep proportion, fewer REM cycles, and higher AHI. PSQI scores were significantly and positively associated with depression risk. These findings provide objective, sleep-based indicators for the early identification and intervention of moderate depressive disorder.

Patients with moderate depressive disorder exhibited significantly lower sleep quality compared to healthy controls, as evidenced by both subjective (PSQI) and objective (PSG) assessments. Consistent with previous reports [28,29], the present results support the hypothesis that depression is a sleep disorder. Sleep fragmentation, as characterized by increased awakenings and disrupted sleep continuity, represents a core PSG feature of depression [30,31]. Patients with moderate depressive disorder had higher PSQI scores than controls, similar to the results of previous studies [28,29]. The PSG results in the present study are largely consistent with the classical sleep model of depression, where sleep fragmentation is a core PSG feature of depression, with an increased proportion of awakenings and disrupted sleep continuity in depressed patients [30,31]. In the present study, increased wakefulness time, ArI, and AHI in patients with moderate depressive disorder suggest a severe disruption of the sleep continuum, possibly contributing to the development of the disorder. Frequent awakenings may have contributed to the decrease in REM sleep time recorded by PSG. Fragmented sleep impairs the functional connectivity of the default mode network (DMN), resulting in sustained cortical hyperarousal and default mode network hyperconnectivity during sleep [32]. This network-level dysfunction impairs emotion regulation and self-emotion processing, thereby increasing susceptibility to depressive symptoms, which may contribute to depression. Frequent awakenings may exacerbate affective dysregulation by further reducing REM sleep duration by shortening sleep cycles [33].

Two findings in the present study diverge from the classical sleep model of depression. Although neither reached statistical significance, the observed trends warrant discussion. First, contrary to the well-established finding of increased REM sleep in depression [34], our moderate-depression group showed a reduced proportion of REM sleep without a concomitant shortening of REM latency. Second, N3 sleep was unexpectedly higher in the depression group, albeit slightly. These discrepancies may be explained by the specific characteristics of our sample. Patients with depression typically present with insufficient sleep, including early awakenings and frequent nocturnal arousals. In contrast, atypical symptoms—predominantly hypersomnia and persistent fatigue—are more prevalent among young adults [35,36]. The majority of participants in the present study were young adults with a high proportion of atypical features. Given that N3 sleep declines significantly with age and that younger individuals possess a higher N3 baseline [37,38], this age effect may mask the disease-related reduction in N3 sleep, yielding a slightly elevated N3 proportion. Beyond the age confound, the atypical symptom profile of our sample implicates distinct pathophysiological mechanisms. Atypical depressive disorder is closely linked to circadian rhythm and neurotransmitter dysregulation [39], with key pathological features including delayed melatonin secretion and altered expression of core circadian genes [40]. Dysregulated circadian rhythms increase depression risk; abnormal melatonin profiles are directly associated with the hypersomnia phenotype, as delayed melatonin secretion prolongs total sleep time and permits full accumulation of slow-wave homeostatic pressure. Core circadian genes promote N3 sleep generation by modulating sleep homeostatic drive [37]. Their altered expression shifts the melatonin phase backward while simultaneously enhancing slow-wave pressure, thereby contributing to hypersomnia [35], prolonged REM latency, and a relatively higher N3 proportion. The absence of shortened REM latency in our sample further supports a mechanism distinct from typical depression. Shortened REM latency in classical depression has been attributed primarily to REM disinhibition resulting from hypofunction of the serotonergic and noradrenergic systems [41]. In atypical depression, however, the neurotransmitter dysregulation pattern appears to differ, with the pathology predominantly involving dysfunction of the dopaminergic and orexin systems [40]. Hyperactivation of the serotonergic system in atypical depressive disorder may attenuate the inhibition of N3 sleep [42], whereas impaired GABAergic transmission can suppress REM sleep. These mechanisms, compounded by subjective sleep prolongation secondary to reduced sleep efficiency, may account for the relative increase in N3 sleep and decrease in REM sleep observed here. Finally, the high proportion of female participants warrants consideration. Women consistently exhibit a higher N3 ratio, likely mediated by estrogen-dependent modulation of sleep architecture [43]. Estrogen enhances serotonergic inhibition of REM sleep and potentiates GABAergic facilitation of N3 sleep. These sex-specific mechanisms may further explain the deviation from the classical depression sleep model observed in the present study.

The above findings highlight the variation in PSG characteristics across the spectrum of depression severity, and suggest the classical REM-dominated model may not be fully applicable to moderate depression. The abnormality of REM sleep parameters may not be the core feature of moderate depression. Future studies should incorporate a longitudinal design to track the dynamic changes of REM parameters during the transition from moderate depression to severe depression, thereby enabling further exploration of sensitive biomarkers in patients with moderate depressive disorder.

### Limitations

This study has several limitations. First, the sample was comprised predominantly of young adults with moderate depression, limiting the generalizability of findings to other age groups or to more severe cases. Second, the cross-sectional design precludes the establishment of causal relationships between sleep disturbances and depression. Third, PSG measurements may be influenced by laboratory-induced sleep alterations. Fourth, cultural specificity and reliance on the HAMD scale may further restrict applicability. Notably, sex differences in sleep architecture and the AHI constitute a critical limitation. Given the limited proportion of male participants in this study, the ROC-derived cutoff values may not be directly generalizable to male populations, and these thresholds should be interpreted with caution. Future validation in gender-specific cohorts is warranted. Finally, the mechanistic pathways linking sleep parameters to depression pathophysiology remain unclear, and longitudinal, multimodal investigations are needed to address these gaps.

## 5. Conclusions

Our results suggest that sleep quality is significantly worse in patients with moderate depression, as observed by both subjective (PSQI) and objective (PSG) measures. PSQI scores were significantly higher in the depression group than in the control group, and significant between-group differences were also observed in TST, WASO, SE, W%, ArI, and REM cycles. The AHI was significantly higher in the moderate depression group than in the control group. However, after adjusting for potential confounders in multivariate binary logistic regression, only the PSQI score remained independently associated with moderate depression. Other PSG parameters did not show significant independent associations. These findings suggest that subjective sleep quality assessed by the PSQI is a robust correlate of moderate depression, whereas the independent utility of individual objective PSG indicators may be limited. Additional studies incorporating larger sample sizes and longitudinal designs are needed to further clarify the relationship between sleep architecture and moderate depression.

## Data Availability

The datasets analyzed during this study and the photos used can be acquired from the corresponding author upon reasonable request.
